# Kinesin Family member 4A: A Potential Predictor for Progression of Human Oral Cancer

**DOI:** 10.1371/journal.pone.0085951

**Published:** 2013-12-30

**Authors:** Yasuyuki Minakawa, Atsushi Kasamatsu, Hirofumi Koike, Morihiro Higo, Dai Nakashima, Yukinao Kouzu, Yosuke Sakamoto, Katsunori Ogawara, Masashi Shiiba, Hideki Tanzawa, Katsuhiro Uzawa

**Affiliations:** 1 Department of Clinical Molecular Biology, Graduate School of Medicine, Chiba University, 1-8-1 Inohana, Chiba, Japan; 2 Department of Dentistry and Oral-Maxillofacial Surgery, Chiba University Hospital, 1-8-1 Inohana, Chiba, Japan; 3 Department of Medical Oncology, Graduate School of Medicine, Chiba University, 1-8-1 Inohana, Chiba, Japan; The University of Hong Kong, China

## Abstract

**Background:**

Kinesin family member 4A (KIF4A), a microtubule-based motor protein, was implicated in regulation of chromosomal structure and kinetochore microtubule dynamics. Considering the functions of KIF4A, we assumed that KIF4A is involved in progression of oral squamous cell carcinomas (OSCCs) via activation of the spindle assembly checkpoint (SAC). However, little is known about the relevance of KIF4A in the behavior of OSCC. We investigated the KIF4A expression status and its functional mechanisms in OSCC.

**Methods:**

The KIF4A expression levels in seven OSCC-derived cells were analyzed by quantitative reverse transcriptase-polymerase chain reaction and immunoblotting analyses. Using a KIF4A knockdown model, we assessed the expression of (SAC)-related molecules (BUB1, MAD2, CDC20, and cyclin B1), cell-cycle, and cellular proliferation. In addition to *in* *vitro* data, the clinical correlation between the KIF4A expression levels in primary OSCCs (n = 106 patients) and the clinicopathologic status by immunohistochemistry (IHC) also were evaluated.

**Results:**

KIF4A mRNA and protein were up-regulated significantly (*P* < 0.05) in seven OSCC-derived cells compared with human normal oral keratinocytes. In the KIF4A knockdown cells, SAC activation was observed via increased BUB1 expression on the kinetochores, appropriate kinetochore localization of MAD2, down-regulation of CDC20, up-regulation of cyclin B1, and cell-cycle arrested at G2/M phase. The results showed that cellular proliferation of KIF4A knockdown cells decreased significantly (*P* < 0.05) compared with control cells. IHC showed that KIF4A expression in primary OSCCs was significantly (*P* < 0.05) greater than in the normal oral counterparts and that KIF4A-positive OSCCs were correlated closely (*P* < 0.05) with tumoral size.

**Conclusions:**

Our results proposed for the first time that KIF4A controls cellular proliferation via SAC activation. Therefore, KIF4A might be a key regulator for tumoral progression in OSCCs.

## Introduction

The kinesin superfamily proteins (KIFs), classified into 14 subfamilies, are ATP-dependent motor proteins with microtubule-dependent plus-end motion ability [1,2]. During mitosis, the activities of KIFs on the spindle microtubule are controlled precisely to ensure that mitotic events are orchestrated in the correct order throughout mitosis [3,4]. These proteins in the interpolar microtubules control a balance of outward forces and inward forces to ensure chromosome capture and attachment to the spindles and prevent spindle elongation before anaphase [5,6]. Among the KIFs, KIF4A controls spindle organization, chromosome alignment, and kinetochore microtubule dynamics with a protein regulator of cytokinesis 1 [4,7-13]. Dysregulation of KIF4A induces abnormal spindle separation and causes aneuploidy of daughter cells [14,15]. Cells affected by aneploidy are characterized by gain or loss of genetic material. They are strongly suspected to be associated with cancer progression [16]. Therefore, we hypothesized that KIF4A might be associated with cancer progression.

The spindle assembly checkpoint (SAC) monitors interactions between kinetochores and spindle microtubules during mitosis and controls metaphase-anaphase transition until all chromosomes establish biorientation. Therefore, the SAC has an important role in cellular proliferation via a cell-cycle control mechanism, which is especially crucial for the accuracy of chromosome segregation [17-19]. Proper functioning of the SAC requires the concerted action of several checkpoint proteins, i.e., BubR1, Bub1, Bub3, Mad1, and Mad2 [19-22]. The active checkpoint inhibits anaphase promoting complex/cyclosome (APC/C) arrest at the anaphase [23-26]. Inhibition of APC/C prevents degradation of several key mitotic proteins, which must be degraded for anaphase to start [23-28]. The presence of unattached chromosomes or a lack of spindle tension that is normally generated by bipolar chromosome attachment results in continued checkpoint activation, mitotic arrest, and eventually programmed cell death [17-19,23-25]. In addition, the SAC has been reported to be defective in a number of human cancers, including oral, colorectal, thyroid, and ovarian cancers, and it is associated with cancer progression [29-32]. 

Because the relationship between the SAC and KIF4A is just beginning to be understood, we assumed that dysregulation of KIF4A is involved in the progression of oral squamous carcinomas (OSCCs) via activation of the SAC [4,5,17,33,34]. We report here that aberrant expression of KIF4A in OSCCs was functionally and clinically linked to tumoral growth and that KIF4A might be a molecular marker for progression of OSCCs.

## Materials and Methods

### Ethics Statement

The Ethical Committee of Graduate School of Medicine, Chiba University approved the study protocol (approval number, 236). The study was performed in accordance with the ethical standards of the Declaration of Helsinki. All patients provided written informed consent.

### OSCC-derived cellular lines and tissue specimens

Immortalized human OSCC-derived cell lines (HSC-2, HSC-3, HSC-4, Ca9-22, Sa3, HO-1-u-1, and KON) were obtained from the Human Science Research Resources Bank (Osaka, Japan) or the RIKEN BRC (Ibaraki, Japan) through the National Bio-Resource Project of the Ministry of Education, Culture, Sports, Science and Technology (MEXT) (Tokyo, Japan). Short tandem repeat proﬁles conﬁrmed cellular identity. All OSCC-derived cells were grown in Dulbecco’s modified Eagle medium/F-12 HAM (Sigma-Aldrich, St. Louis, MO) supplemented with 10% fetal bovine serum (Sigma) and 50 units/ml penicillin and streptomycin (Sigma). Primary cultured human normal oral keratinocytes (HNOKs) were used as a normal control [35-40]. They were healthy oral mucosa epithelium specimens collected from young patients at Chiba University Hospital. Three independent HNOKs were primary cultured and maintained in Oral Keratinocyte Medium (ScienCell Research Laboratories, Carlsbad, CA) comprised of 5 ml of oral keratinocyte growth supplement (ScienCell Research Laboratories) and 5 ml of penicillin/streptomycin solution (ScienCell Research Laboratories). 

One hundred six primary OSCC samples and patient-matched normal epithelium were obtained during surgeries performed at Chiba University Hospital. The resected tissues were fixed in 20% buffered formaldehyde solution for pathologic diagnosis and immunohistochemistry (IHC). Histopathologic diagnosis of each OSCC samples was performed according to the World Health Organization criteria by the Department of Pathology of Chiba University Hospital [41]. Clinicopathologic staging was determined by the TNM classification of the International Union against Cancer [42]. All patients had OSCC that was histologically confirmed, and tumoral samples were checked to ensure that tumoral tissue was present in more than 80% of specimens.

### Preparation of cDNA and protein

Total RNA was isolated using Trizol Reagent (Invitrogen, Carlsbad, CA) according to the manufacturer’s instructions. cDNA was generated using 5 µg total RNA from OSCC-derived cell lines using Ready-To-Go You-Prime First-Strand Beads (GE Healthcare, Buckinghamshire, UK) and oligo (dT) primer (Hokkaido System Science, Sapporo, Japan) according to the manufacturer’s instructions. The cells were washed twice with cold phosphate-buffered saline (PBS) and centrifuged briefly. The cell pellets were incubated at 4°C for 30 minutes in a lysis buffer (7 M urea, 2 M thiourea, 4% w/v CHAPS, and 10 mM Tris pH 7.4) with a proteinase inhibitor cocktail (Roche, Diagnostics). The protein concentration was measured with BCA Protein Assay Kit (Thermo, Rockford, IL).

### mRNA expression analysis

Real-time quantitative reverse transcriptase-polymerase chain reaction (qRT-PCR) was performed using LightCycler 480 apparatus (Roche Diagnostics, Mannheim, Germany). Primers and universal probes were designed using the Universal Probe Library (Roche Diagnostics) which speciﬁes the most suitable set. The primer sequences used for qRT-PCR were: *KIF4A*, forward, *5′-TCTGTTTCAGGCTGCTTTCA -3′*; reverse, *5′-GCCCTG AAATATTTGATTGGAG -3′*; and universal probe 25, and the glyceraldehyde-3-phosphate dehydrogenase (GAPDH) , forward, *5′-CATCTCTGCCCCCTCTGCTGA-3′*; reverse, *5′-GGATGACCTTGCCCACAGCCT-3′*; and universal probe 60. The transcript amount for *KIF4A* was estimated from the respective standard curves and normalized to *GAPDH* transcript amount determined in corresponding samples. All samples were analyzed in triplicate and three independent preparations of RNA were analyzed from each cell line.

### Immunoblotting analysis

Protein extracts (20 µg) were separated by sodium dodecyl sulfate polyacrylamide gel electrophoresis in 4–12% gel, transferred to nitrocellulose membranes, and blocked for 1 hour at room temperature in Blocking One (Nacalai Tesque, Tokyo, Japan). The membranes were incubated with rabbit anti-KIF4A polyclonal antibody (Gene Tex, San Antonio, TX), mouse anti-GAPDH monoclonal antibody (Santa Cruz Biotechnology, Santa Cruz, CA), mouse anti-CDC20 monoclonal antibody (Santa Cruz Biotechnology), and rabbit anti-cyclin B1 polyclonal antibody (Cell Signaling Technology, Danvers, MA) overnight at 4°C. The membranes were washed with 0.1% Tween-20 in Tris-buffered saline, incubated with secondary antibody and coupled to horseradish peroxidase-conjugated anti-rabbit or anti-mouse IgG (Promega, Madison, WI) for 1 hour at room temperature. Finally, the membranes were detected using SuperSignal West Pico Chemiluminescent substrate (Thermo), and immunoblotting was visualized by exposing the membranes to ATTO Light-Capture II (ATTO, Tokyo, Japan). Signal intensities were quantitated using the CS Analyzer version 3.0 software (ATTO).

### Transfection with shRNA plasmid

OSCC cellular lines (HSC-3 and Ca9-22) were transfected with KIF4A shRNA (shKIF4A) or control shRNA (shMock) vectors (Santa Cruz Biotechnology) using Lipofectamine LTX and Plus Reagents (Invitrogen). After transfection, the stable transfectants were isolated by the culture medium containing 2 ng/mL Puromycin (Invitrogen). Two to three weeks after transfection, viable colonies were transferred to new dishes. shKIF4A and shMock cells were used for further experiments.

### Immunofluorescence

The transfectants were plated on chamber slides (BD Falcon, Franklin Lakes, NJ) at 50% confluency, washed with ice-cold PBS, and fixed with 4% paraformaldehyde-PBS for 20 minutes, then permeabilized in PBS containing 0.2% Triton X-100 as previously described [43]. We used the following primary antibodies to detect SAC activation: rabbit anti-KIF4A polyclonal antibody (Gene Tex), mouse anti-BUB1 monoclonal antibody (Santa Cruz Biotechnology), and mouse anti-MAD2 antibody (Santa Cruz Biotechnology). Fixed cells were incubated with a blocking solution containing 0.5% bovine serum albumin for 1 hour at room temperature and incubated with primary antibodies overnight at 4°C. After rinsing with PBS, cells were incubated with secondary antibodies for 1 hour at 37°C. The secondary antibodies were fluorescein isothiocyanate-conjugated anti-rabbit IgG antibody (Vector Laboratories, Burlingame, CA) and Texas-Red-conjugated anti-mouse IgG antibody (Vector Laboratories) incubated for 1 hour at room temperature in the dark. Finally, the sections were washed three times with PBS and mounted using Mounting Medium with DAPI (Vector Laboratories). The immunofluorescence was performed by confocal microscopy and analyzed with the Fluoview software (Olympus Optical, Tokyo, Japan).

### Cell-cycle analysis

To synchronize cells at the G0/G1 or G2/M transition, the cells were deprived of serum for 48 hours or treated with 200 ng/ml nocodazole (Sigma) for 20 hours [44,45]. To determine the cell-cycle distribution, the cells were harvested, washed with PBS, and probed with CycleTEST Plus DNA reagent kit (Becton-Dickinson, San Jose, CA) according to the manufacturer's protocol. Flow cytometric determination of DNA content was analyzed by BD AccuriTM C6 Flow Cytometer (Becton-Dickinson). The fractions of the cells in the G0-G1, S, and G2-M phases were analyzed using FlowJo software (Tree Star, Ashland, OR).

### Cellular growth

The transfectants were seeded in six-well plates at a density of 1 × 10^4^ viable cells per well. Cells were harvested for 168 hours, and counted every 24 hours. At the indicated time points, the cells were trypsinized and counted using a hemocytometer in triplicate samples.

### IHC

IHC was performed on 4-µm sections of paraffin-embedded specimens using rabbit anti-KIF4A polyclonal antibody (Gene Tex). Briefly, after deparaffinization and hydration, the endogenous peroxidase activity was quenched by 30 minutes incubation in a mixture of 0.3% hydrogen peroxide solution in 100% methanol; the sections were blocked for 2 hours at room temperature with 1.5% blocking serum (Santa Cruz Biotechnology) in PBS before reacting with anti-KIF4A antibody at 4°C in a moist chamber overnight. Upon incubation with the primary antibody, the specimens were washed three times in PBS and treated with Envision reagent (DAKO, Carpinteria, CA) followed by color development in 3,3’-diaminobenzidine tetrahydrochloride (DAKO). The slides then were slightly counterstained with hematoxylin, dehydrated with ethanol, cleaned with xylene, and mounted. Nonspecific binding of an antibody to proteins other than the antigen sometimes occurred. As a negative control, triplicate sections were immunostained without exposure to primary antibodies, which confirmed the staining specificity (Figure S1A). To quantify the status of KIF4A protein expression in those components, we used the IHC scoring systems described previously [39,40,46-49]. In summary, the mean percentages of positive tumoral cells were determined in at least three random fields at 400× magnification in each section. The intensity of the KIF4A-immunoreaction was scored as follows: 0+, none; 1+, weak; 2+, moderate; and 3+, intense. The cellular number and the staining intensity were multiplied to produce a KIF4A IHC score. We considered the staining intensity of the skeletal muscle cells, which were positive controls for KIF4A (Figure S1B). Cases with a KIF4A IHC score exceeding 95.0 (+3 standard deviation [SD] score for normal tissue) were defined as KIF4A-positive. The ±3-SD cutoff, which statistically just 0.2% of the measurement would be expected to fall outside this range, was used because it was unlikely to be affected by random experimental error produced by sample manipulation [50]. Two independent pathologists from Chiba University Hospital, neither of whom had any knowledge of the patients’ clinical status, made these judgments.

### Statistical analysis

In comparisons of KIF4A expression levels, statistical significance was evaluated using the Mann-Whitney's U test. Relationships between KIF4A-immunohistochemical staining scores and clinicopathological profiles were evaluated by χ^2^ test, Fisher's exact test, and Mann-Whitney's U test. *P* < 0.05 was considered significant. The data are expressed as the mean ± standard error of the mean (SEM).

## Results

### Evaluation of KIF4A expression in OSCC-derived cellular lines

**Figure 1:**
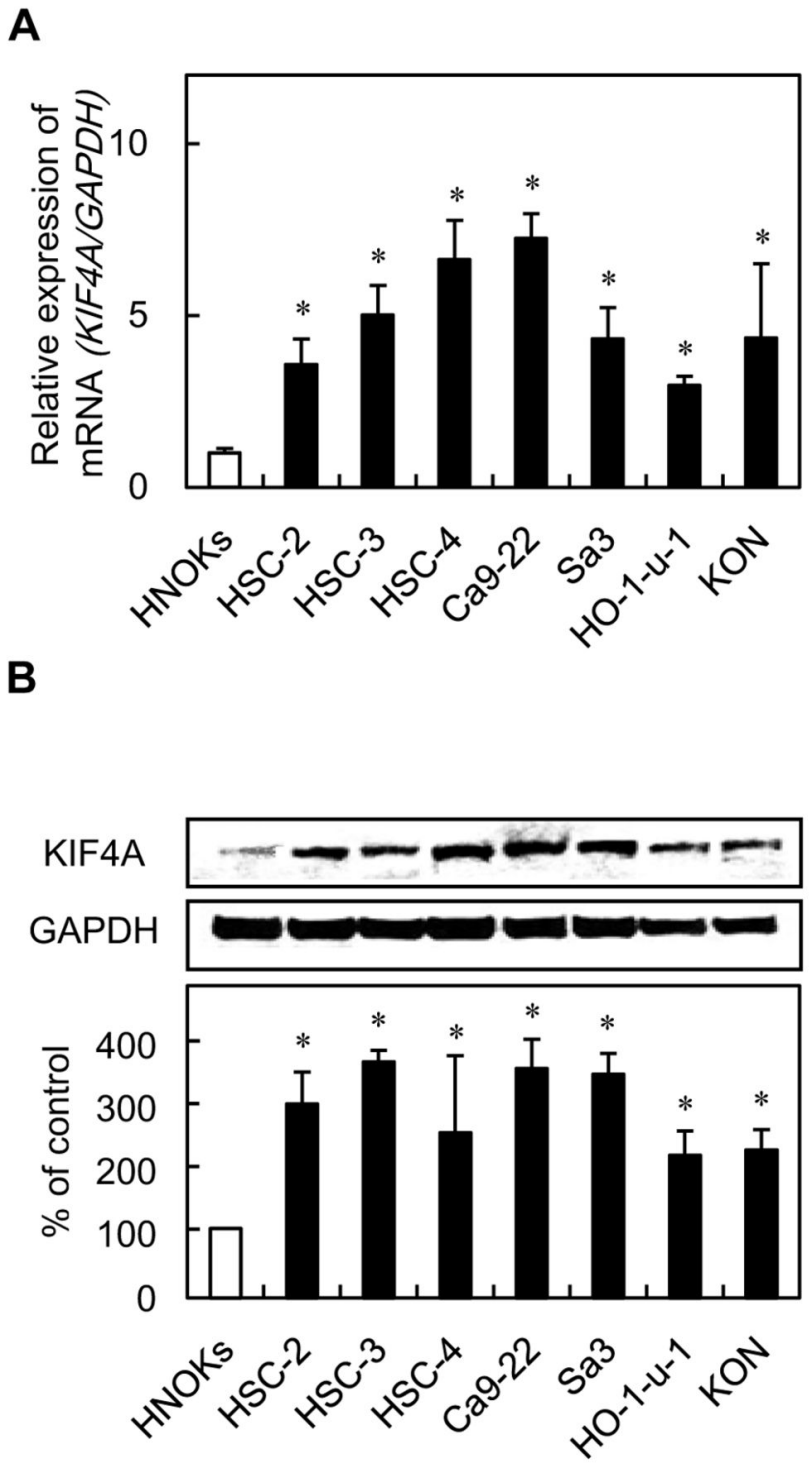
Evaluation of KIF4A expression in OSCC-derived cellular lines. (**A**) Quantification of KIF4A mRNA expression in OSCC-derived cellular lines by qRT-PCR analysis. Significant up-regulation of KIF4A mRNA is seen in seven OSCC-derived cellular lines compared with the HNOKs (*P* < 0.05, Mann-Whitney U test). Data are expressed as the means ± SEM of triplicate results. (**B**) Immunoblotting analysis of KIF4A protein in the OSCC-derived cellular lines and HNOKs. KIF4A protein expression is up-regulated in the OSCC-derived cellular lines compared with that in the HNOKs. Densitometric KIF4A protein data are normalized to GAPDH protein levels. The values are expressed as a percentage of the HNOKs (*P* < 0.05, Mann-Whitney U test).

To investigate the mRNA expression of *KIF4A*, we performed qRT-PCR analysis using seven OSCC-derived cellular lines (HSC-2, HSC-3, HSC-4, Ca9-22, Sa3, Ho-1-u-1, and KON) and HNOKs. *KIF4A* mRNA was up-regulated significantly in all OSCC-derived cellular lines compared with the HNOKs (Figure 1A). Data are expressed as the mean ± SEM of triplicate results (*P* < 0.05). We also performed immunoblotting analysis to investigate KIF4A protein expression status in the OSCC-derived cellular lines and the HNOKs (Figure 1B). A significant increase in KIF4A protein expression was observed in all OSCC cellular lines compared with the HNOKs. Expression analysis indicated that both transcription and translation products of this molecule were highly expressed in OSCC-derived cellular lines.

### Establishment of KIF4A knockdown cells

**Figure 2:**
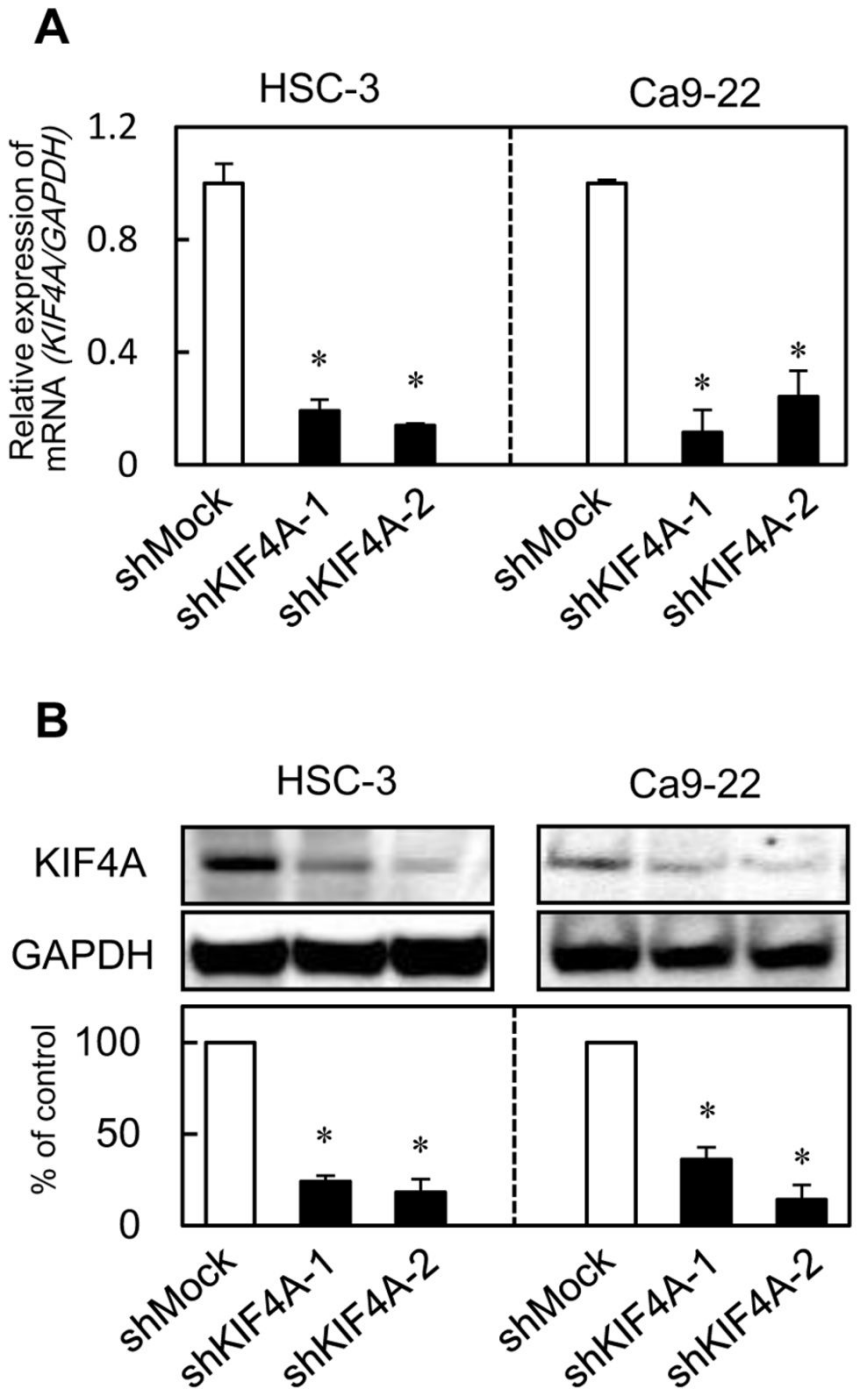
Expression of KIF4A in shKIF4A cells. (**A**) qRT-PCR shows that KIF4A mRNA expression in the shKIF4A cells (HSC-3-and Ca9-22-derived transfectants; 2 clones each) is significantly lower than in the shMock cells (*P* < 0.05, Mann-Whitney U test). (**B**) Immunoblotting analysis shows that the KIF4A protein levels in shKIF4A-transfected cells (HSC-3- and Ca9-22-derived transfectants; 2 clones each) also have decreased markedly compared with that in the shMock cells (*P* < 0.05, Mann-Whitney U test).

Since KIF4A expression was up-regulated in the OSCC cellular lines, we established KIF4A knockdown cells using shRNA technologies. HSC-3 and Ca9-22 cells were transfected with KIF4A shRNA (shKIF4A) and the control shRNA (shMock) plasmids. Expression levels of KIF4A mRNA and protein in the shKIF4A cells were significantly lower than those in the shMock cells (HSC-3 and Ca9-22-derived transfectants) (Figure 2A, B) (*P* < 0.05).

### Activation of the SAC

**Figure 3:**
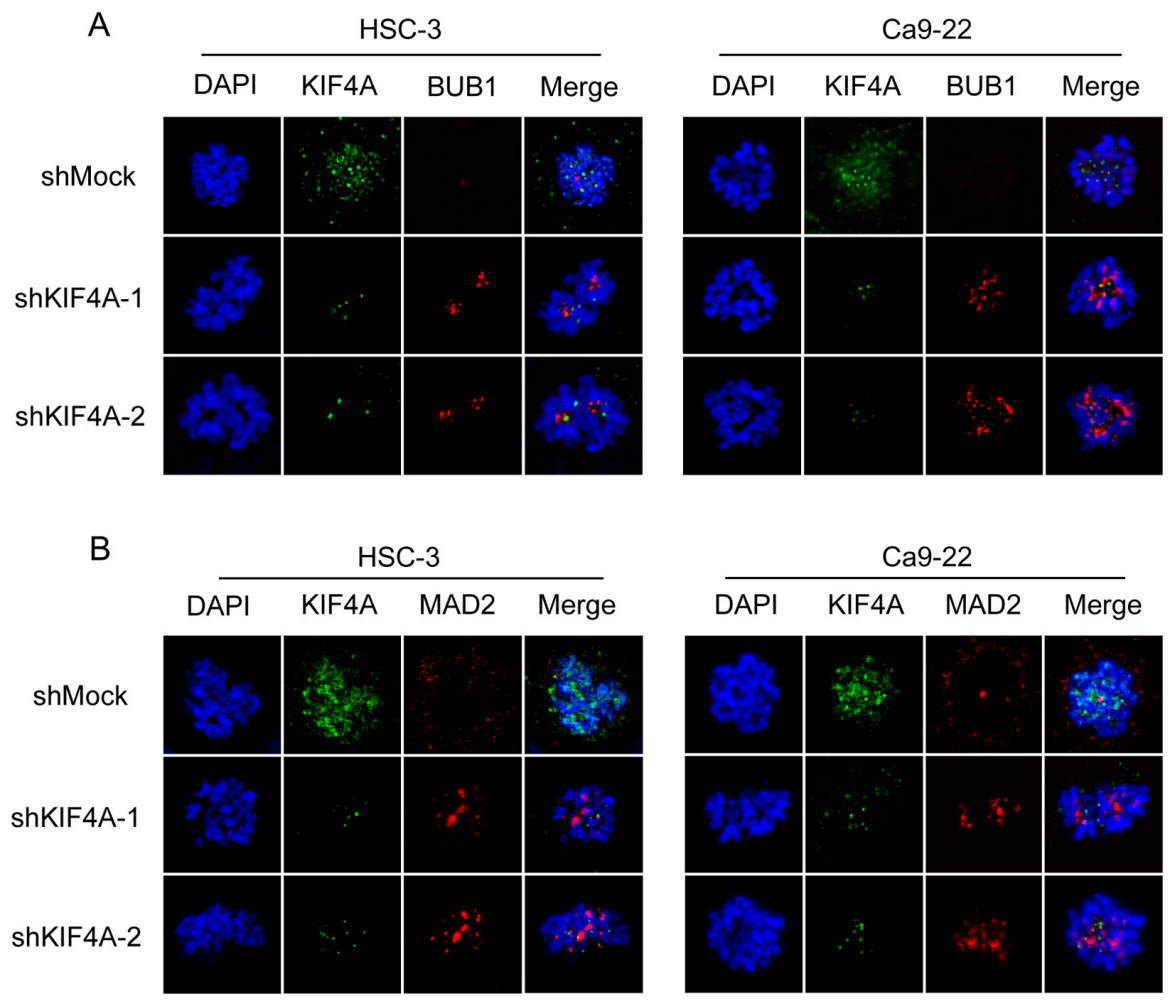
Localization of BUB1 and MAD2 during the prometaphase in shMock and shKIF4A cells. Localization of BUB1 and MAD2 to the kinetochores is compared in shKIF4A and shMock cells by immunofluorescence. (**A**) BUB1 on kinetochores increased in shKIF4A cells compared with that in the shMock cells (green, KIF4A; red, BUB1; blue, DNA). (**B**) Appropriate localization of MAD2 is seen in shKIF4A cells, whereas kinetochore localization is not seen in the shMock cells (green, KIF4A; red, MAD2; blue, DNA).

**Figure 4:**
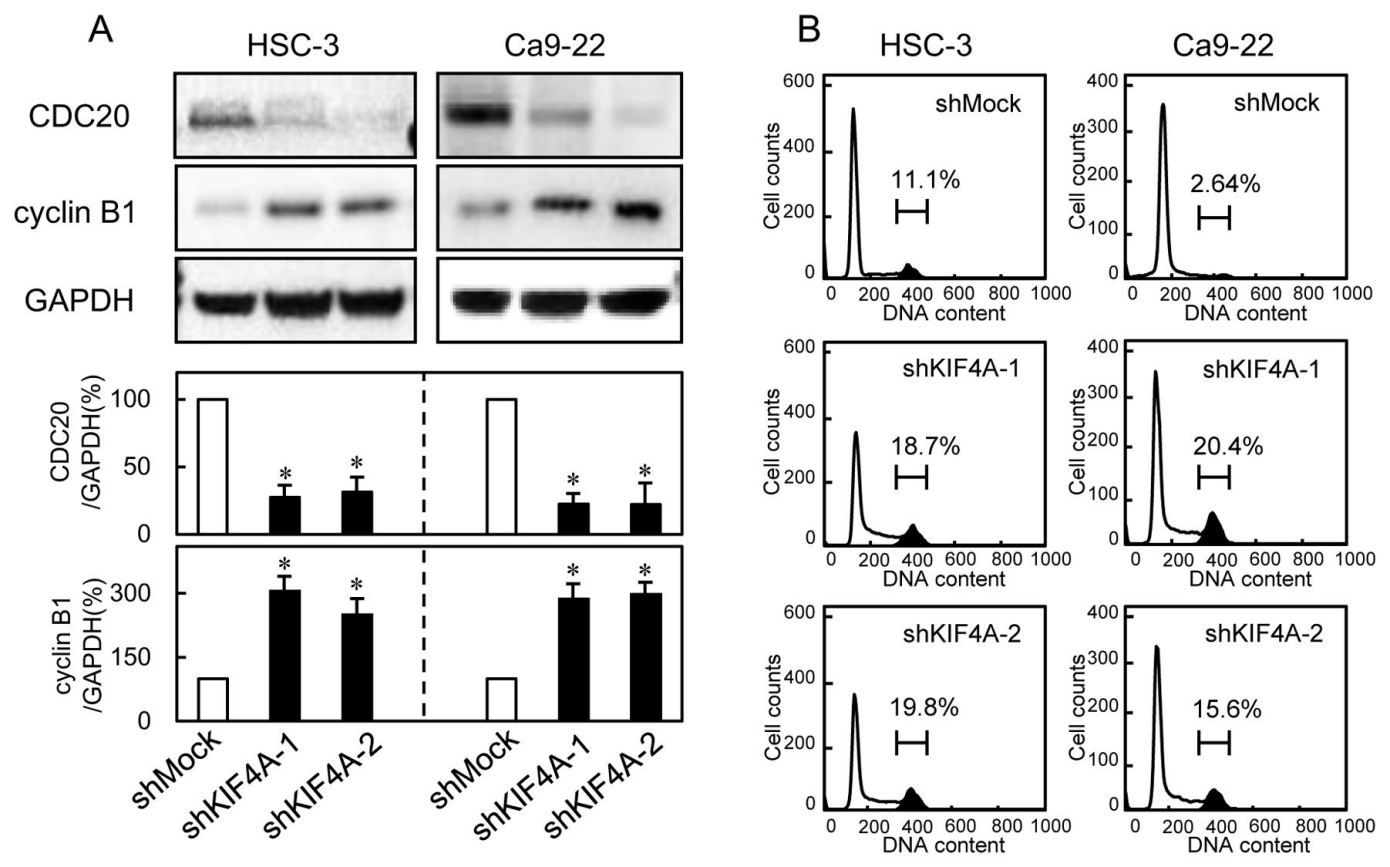
shKIF4A promote G2/M arrest via SAC activation. To investigate SAC activation and cell-cycle progression, we determined the expression levels of CDC20 and cyclin B1, and DNA content in the G0-G1, S, and, G2-M phases. (**A**) shKIF4A cells showed down-regulation of CDC20 and up-regulation of cyclin B1 (HSC-3- and Ca9-22-derived transfectants; 2 clones each) compared with shMock cells (*P* < 0.05, Mann-Whitney U test). (**B**) Flow cytometric analysis was performed to investigate the cell cycle in shKIF4A and shMock cells. The percentage of the G2/M phase in shKIF4A cells (HSC-3- and Ca9-22-derived transfectants; 2 clones each) has increased markedly compared with shMock cells (*P* < 0.05, Mann-Whitney’s U test).

To investigate the mechanism by which KIF4A is related to the SAC, we performed immunofluorescence analysis for the spindle-checkpoint protein (BUB1) and the checkpoint sensor protein (MAD2). BUB1 was found in condensed chromosomes in the shKIF4A cells but was not localized on kinetochores in the shMock cells (Figure 3A). MAD2 was localized on kinetochores in the shKIF4A cells, whereas kinetochore localization was not seen in the shMock cells (Figure 3B). The direct target of SAC activation is CDC20, which is the activator of the anaphase-promoting complex/cyclosome (APC/C) [23-26]. Moreover, cyclin B1 is a critical regulator of G2/M progression and M-G1 transition [27]. To evaluate the M phase arrest by activation of the SAC, we also performed immunoblotting of CDC20 and cyclin B1, and flow cytometric analysis. As expected, while CDC20 was significantly down-regulated, cyclin B1 was up-regulated in shKIF4A cells (Figure 4A). Furthermore, the percentage of the G2/M phase in shKIF4A cells was significantly (P < 0.05) higher than that in shMock cells (Figure 4B). These results indicated that down-regulation of KIF4A might induce cell-cycle arrest or delay in the M phase via SAC activation.

### Reduced cellular growth in KIF4A knockdown cells

**Figure 5:**
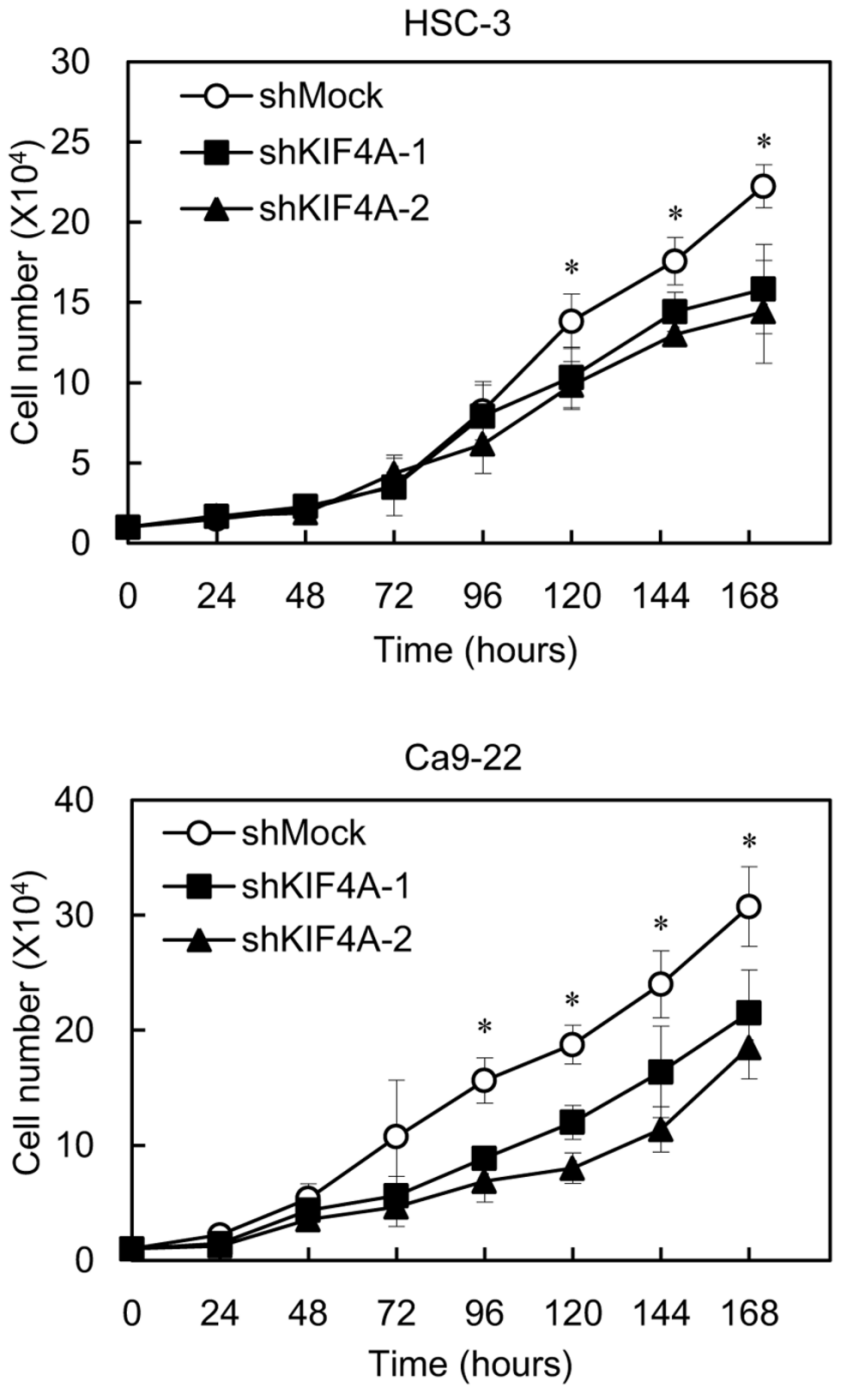
Reduced cellular growth in shKIF4A cells. To determine the effect of shKIF4A on cellular proliferation, shKIF4A and shMock cells were seeded in six-well plates at a density of 1×10^4^ viable cells/well. Both transfectants were counted on seven consecutive days. The cellular growth of shKIF4A-transfected cells (HSC-3- and Cas9-22-derived transfectants; 2 clones each) were significantly inhibited compared with shMock cells after 7 days (168 hours). The results were expressed as the means ± SEM of values from three assays. The asterisks indicate significant differences between the shKIF4A and shMock cells (*P* < 0.05, Mann-Whitney U test).

To evaluate the effect of KIF4A knockdown on cellular growth, we performed a cellular proliferation assay. shKIF4A and shMock cells were seeded in six-well plates at a density of 1 × 10^4^ viable cells/well that were counted for 168 hours. There was a significant (*P* < 0.05) decrease in cellular growth of the shKIF4A cells compared with the shMock cells (Figure 5).

### Evaluation of KIF4A protein expression in primary OSCC

**Figure 6:**
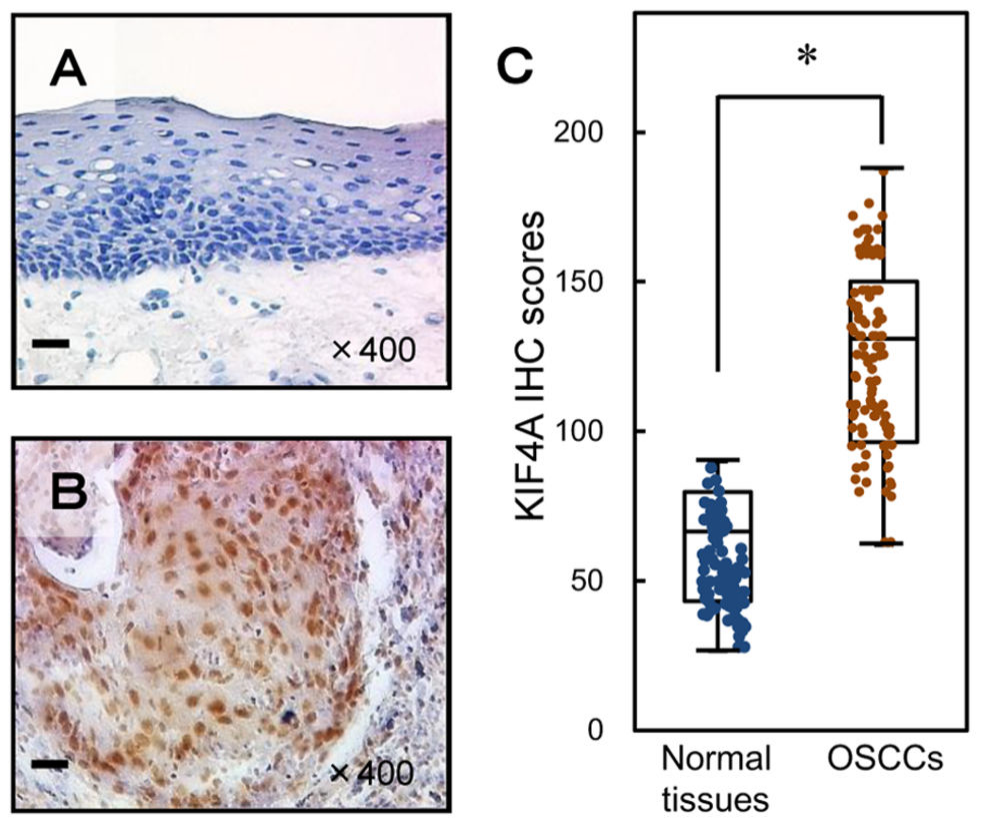
Evaluation of KIF4A protein expression in primary OSCCs. Representative IHC results for KIF4A protein in normal oral tissue (**A**) and primary OSCC (**B**). **A**, **B**: Original magnification, ×400. Scale bars, 10 μm. Strong KIF4A immunoreactivity was detected in primary OSCCs; normal oral tissues show almost negative immunostaining. **C**: The state of KIF4A protein expression in primary OSCCs (n=106) and the normal counterparts. The KIF4A IHC scores were calculated as follows: IHC score = 0× (number of unstained cells in the field) +1× (number of weakly stained cells in the field) +2× (number of moderately stained cells in the field) +3× (number of intensely stained cells in the field). The KIF4A IHC scores for normal oral tissues and OSCCs ranged from 26.5 to 103 (median, 66.5) and 62.3 to 188 (median, 131), respectively. KIF4A protein expression levels in OSCCs are significantly (*P* < 0.05, Mann-Whitney’s U test) higher than in normal oral tissues.

Representative IHC results for KIF4A protein in normal oral tissue and primary OSCC are shown in Figure 6A-C. Positive staining was seen predominantly in the nuclei of primary OSCC samples (Figure 6A, B). The KIF4A IHC scores in primary OSCCs (62.3-188; median, 131) were significantly higher than those in normal tissues (26.5-103; median, 66.5) (Figure 6C) (*P* < 0.05). We defined cases with an IHC score exceeding 95 (+3 SD score for normal tissue) as KIF4A-positive. The correlations between the clinicopathologic characteristics of the patients with OSCC and the status of KIF4A protein expression are shown in Table 1. Among the clinical parameters, KIF4A expression was related significantly (*P* < 0.05) to the primary size of the OSCC tumors. These results suggested that KIF4A might be related closely to tumoral progression. 

**Table 1:** Correlation between KIF4A expression and clinical classification in OSCCs.

		**Results of immunostaining**		
**Clinical classification**		**No. patients (%)**		
	**Total**	**KIF4A- negative**	**KIF4A-positive**	***P* value^*a*^**
Age at surgery (years)				
<60	39	14 (36)	25 (64)	
≧60, <70	22	12 (55)	10 (45)	0.121**^*b*^**
≧70	45	10 (22)	35 (78)	
Gender				
Male	75	29 (39)	46 (52)	0.122**^*c*^**
Female	31	7 (23)	24 (77)	
T-primary tumor				
T1	9	5 (56)	4 (44)	0.005**^*d*^**
T2	58	25 (43)	33 (57)	
T3	19	3 (16)	16 (84)	
T4	20	3 (15)	17 (85)	
T1+T2	67	30 (45)	37 (55)	0.010**^*c*^**
T3+T4	39	6 (15)	33 (85)	
N-regional lymph node				
N (-)	45	15 (33)	30 (67)	0.920**^*c*^**
N (+)	61	21 (34)	40 (66)	
Stage				
I	9	5 (56)	4 (44)	0.121**^*d*^**
II	38	14 (37)	24 (63)	
III	11	4 (36)	7 (64)	
IV	48	13 (27)	35 (73)	
Histopathologic type				
Well	61	23 (38)	38 (62)	0.352**^*d*^**
Moderately	37	11 (30)	26 (70)	
Poorly	8	2 (25)	6 (75)	
Tumoral site				
Gingiva	32	9 (38)	23 (63)	0.861**^*d*^**
Tongue	65	27 (42)	38 (58)	
Buccal mucosa	6	0 (0)	6 (100)	
Oral floor	3	0 (0)	3 (100)	

***^a^*** *P*<0.05 was considered significant, ***^b^***χ^2^ test, ***^c^***Fisher's exact test, ***^d^***Mann-Whitney's U test.

## Discussion

Since KIF4A was overexpressed frequently in OSCC-derived cell lines, we established KIF4A knockdown cells to see whether or not KIF4A has critical functions in OSCCs. shKIF4A cells showed decreased cellular growth by SAC activation leading to cell-cycle arrest of the M phase. In addition to the *in vitro* data, we also found that KIF4A was up-regulated in clinical OSCC samples compared with normal tissues and that KIF4A-positive OSCC cases were related closely to tumoral size. These results indicated that KIF4A is linked to regulation of the cell cycle in the M phase and plays an important role in tumoral progression in OSCCs.

Many studies have indicated that KIFs play critical roles, including tumoral development and progression, in cancers [12-14,31,32]. Among them, KIF2C, KIF18A, KIF20A, and KIF20B were up-regulated in several malignancies and associated with tumoral progression [51-56]. In contrast, KIF1A was down-regulated in nasopharyngeal carcinoma and reported as a potential tumoral suppressor [57]. KIF4A was overexpressed in cervical and lung cancers [58,59], whereas KIF4A was down-regulated in gastric cancers [60]. In addition to the current data that KIF4A was significantly up-regulated in OSCCs, Castillo et al. reported that overexpression of some motor kinesins, including KIF4A, which generate additional outward forces during mitosis, induces excessive spindle separation leading to unequal distribution of genetic material in anaphase and eventually development of daughter cells affected by aneuploidy [61]. In contrast, several studies have reported that loss of KIF4A might affect tumoral proliferation and carcinogenesis [34,62]. Because of conflicting results such as these, more studies are needed to better understand the complex roles KIF4A plays in cancer development and progression.

The novel ﬁndings in the current study are the correlation between KIF4A knockdown and disrupted mitosis resulting from SAC activation. The SAC activation pathway is tightly controlled by spindle checkpoint proteins, such as BUB1 and MAD2 [17-22]. In response to inappropriate attachment of chromosomes to spindle microtubules, which cause abnormal cellular division, BUB1 is concentrated at the kinetochores in mitotic cells and helps localize MAD2 on kinetochores [62-64]. Activated MAD2 localized properly on kinetochores can inhibit CDC20, which activates the ubiquitin ligase known as APC/C [65-67]. Thus, this evidence indicates that KIF4A knockdown might cause SAC activation through APC/C inactivation [23-27]. 

We found that high levels of cyclin B1 and M phase arrest were observed in the KIF4A knockdown cells. During induction of G2/M phase arrest after treatment of cells with microtubule inhibitors which activate the SAC by interfering with microtubule dynamics and stability, such as nocodazole and paclitaxel, a marked increase in cyclin B1 protein levels also was seen [65-69]. Therefore, down-regulation of KIF4A induced the cell cycle arrest of OSCC cells by similar functions of the microtubule inhibitors.

KIF4A protein expression levels in primary OSCCs were correlated with tumoral size; this strongly implies that KIF4A may play an important role in OSCCs progression and development. Taken together, stratification of patients with OSCC based on KIF4A status may provide a more personalized approach to human oral cancer therapy.

## Conclusion

The current results showed for the ﬁrst time that KIF4A is overexpressed frequently in OSCC, which suggests interference in the function of the spindle checkpoint proteins such as BUB1, MAD2, and CDC20. KIF4A expression was correlated with tumoral size in KIF4A-positive cases, suggesting that SAC activation plays an important role in cellular proliferation in OSCC. KIF4A expression is likely to be a key regulator of tumoral progression in OSCCs.

## Supporting Information

Figure S1 **KIF4A immunoactivity in negative and positive controls.** To evaluate the specificity of KIF4A antibody, we examined the staining intensity of negative and positive controls. Original magnification, ×400. Scale bars, 10 μm. (**A**) As a negative control, primary OSCC samples were immunostained without exposure to primary antibodies. There were no stained cells. (**B**) Skeletal muscle tissue is a positive control for KIF4A. Strong KIF4A immunoreactivity was specifically detected in the nucleus of cells.
(TIF)
