# Supplementary figures and images for: Kinesin Family member 4A: A Potential Predictor for Progression of Human Oral Cancer
Source: PLoS One. 2013 Dec 30;8(12):e85951. doi: 10.1371/journal.pone.0085951 (PMC3875575; doi:10.1371/journal.pone.0085951)

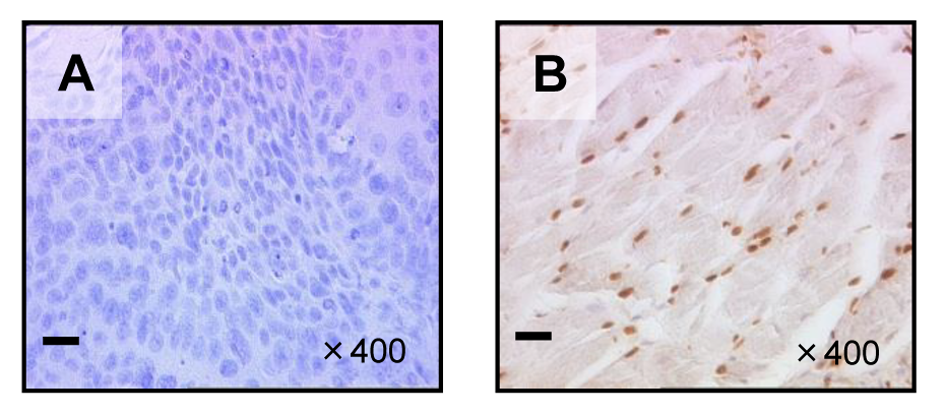

Supplement: Figure S1 — KIF4A immunoactivity in negative and positive controls. To evaluate the specificity of KIF4A antibody, we examined the staining intensity of negative and positive controls. Original magnification, ×400. Scale bars, 10 μm. (A) As a negative control, primary OSCC samples were immunostained without exposure to primary antibodies. There were no stained cells. (B) Skeletal muscle tissue is a positive control for KIF4A. Strong KIF4A immunoreactivity was specifically detected in the nucleus of cells. (TIF) [file pone.0085951.s001.tif]
